# Longitudinal genomic profiling of chemotherapy-related CHIP variants in patients with ovarian cancer

**DOI:** 10.3389/fonc.2025.1538446

**Published:** 2025-04-29

**Authors:** Sara Corvigno, Jun Yao, Amma Asare, Li Zhao, Joseph Celestino, Richard A. Hajek, Ency A. Arboleda Goette, Ridge T. Rogers, Raymond N. Montoya, Ping Song, Qingxiu C. Zhang, Xingzhi Song, Mohammad M. Mohammad, Kenna R. Shaw, Jianhua Zhang, Karen H. Lu, Amir A. Jazaeri, Shannon N. Westin, Anil K. Sood, Sanghoon Lee

**Affiliations:** ^1^ Department of Gynecologic Oncology and Reproductive Medicine, The University of Texas MD Anderson Cancer Center, Houston, TX, United States; ^2^ Department of Molecular & Cellular Oncology, The University of Texas MD Anderson Cancer Center, Houston, TX, United States; ^3^ Department of Genomic Medicine, The University of Texas MD Anderson Cancer Center, Houston, TX, United States; ^4^ Institute for Personalized Cancer Therapy (IPCT) Genomic Laboratory (IPCT Lab), Sheikh Khalifa Bin Zayed Al Nahyan Institute for Personalized Cancer Therapy, The University of Texas MD Anderson Cancer Center, Houston, TX, United States

**Keywords:** oncology, ovarian cancer, biomarkers, clonal hematopoiesis of indeterminate potential (CHIP), t-MDS/AML

## Abstract

**Introduction:**

Clonal hematopoiesis (CH) is characterized by the presence of hematopoietic stem cells (HSCs) with the potential of clonally expanding and giving rise to hematological malignancies. Clonal hematopoiesis of indeterminate potential (CHIP) is the outgrowth of a single HSC clone with an acquired somatic mutation in the absence of hematological abnormalities. CHIP variants occur with a variant allele frequency (VAF) of at least 2% in peripheral blood. This definition does not account for less frequent mutations that give rise to hematopoietic clones. Previous studies indicate an association between CH and secondary hematologic malignancies in cancer patients who receive chemotherapy.

**Methods:**

To discover novel candidate CHIP mutations, including those with extremely low VAFs, we performed an in-depth characterization of low-frequency CHIP variants in a highly selected group of patients with high-grade serous ovarian cancer (HGSC) before and after neoadjuvant chemotherapy (NACT). We performed comprehensive ultra-high-depth whole-exome sequencing of circulating free DNA (cfDNA) and matched white blood cell (WBC) DNA from pre- (n=9) and post-NACT (n=9) samples from HGSC patients who had excellent response (ER; n=4) or poor response (PR; n=5) to NACT.

**Results:**

Variants present in both the WBC DNA and cfDNA from a patient were considered candidate CHIP variants. We identified 93,088 candidate CHIP variants in 13,780 genes. Compared with pre-NACT samples, post-NACT samples tended to have fewer CHIP mutations with VAFs of less than 5%, which may reflect the negative selective pressure of chemotherapy on rare hematopoietic clones. Finally, we identified CHIP variants in tumor samples matched to the liquid biopsies.

**Discussion:**

Our innovative sequencing approach enabled the discovery of a large number of novel low-frequency candidate CHIP mutations, whose frequency and composition are affected by chemotherapy, in the cfDNA of patients with HGSC. The CHIP variants that were enriched after chemotherapy, if validated, might become essential predictive markers for therapy-related myeloid neoplasia.

## Introduction

The clonal expansion of hematopoietic clones occurs when mutations in specific genes confer a selective fitness advantage to specific hematopoietic stem cells. In the absence of signs of hematologic malignancy, this event can be referred to as *clonal hematopoiesis of indeterminate potential* (CHIP), as its impact on the future development of hematological malignancies is unknown. The mutations, or variants, associated with this process are called CHIP mutations. Such mutations occur in the absence of morphological variations in blood cells characteristic of a known hematologic malignancy and were previously defined to have a VAF of 2% or greater in peripheral blood (1). CHIP mutations become more frequent with age. Their frequency increases from 0% in people younger than 40 years to 9.5% in people older than 70 years (2). Several studies established a direct association between certain CHIP mutations and a higher risk of blood cancers (3–5). Some of these studies, using conditional knock-outs of specific genes such as *ASXL2*, whose recurrent mutations are frequently detected in the leukemic cells of patients with myeloid malignancies, successfully modeled the progression of clonal hematopoiesis to malignant transformation *in vivo* (6, 7). *DNMT3A* and *TET2* are also candidate genes for CHIP mutations, as loss-of-function mutations in these genes are commonly detected in the peripheral blood samples of patients with myelodysplastic syndromes and acute myeloid leukemia (8).

Most previous studies seeking to identify CHIP variants used DNA sequencing of a panel of target genes, which mostly included genes associated with leukemia or other hematological malignancies (9). This approach tends to exclude a substantial number of candidate CHIP mutations with extremely low VAFs that can still give rise to malignant hematological clones. Moreover, the heterogeneous clinicopathological characteristics of the cohorts of patients with solid tumors analyzed in prior CHIP studies includes bias related to different type and length of treatments, which represents a major pitfall in this research field. Identifying patterns of change in the frequency and number of multiple CHIP variants, instead of single mutations, represents a more efficient strategy to stratify patients based on their individual risk of developing hematological malignancies. Therefore, the lack of suitable patient cohorts, and well-defined technical approaches for comprehensive identification of all CHIP variants, represent important obstacles to the advancement of this field.

Chemotherapy can drive the expansion of malignant hematological cell clones (10). Clinically and molecularly, therapy-related acute myeloid leukemia (t-AML) and therapy-related myelodysplastic syndrome (t-MDS) are distinct entities, but both are characterized by the frequent rearrangement of chromosomes 5 and 7, an increased incidence of *TP53* mutation, and high rates of resistance to chemotherapy (11). Clonal evolution is a fundamental process in the development of t-MDS and t-AML (12, 13); the proliferation of clones with pre-leukemic founder mutations in epigenetic regulating genes, such as the DNA methyltransferase 3A gene *DNMT3A*, tet oncogene family member 2 gene *TET2*, and additional sex combs-like 1 gene *ASXL1*, due to the accumulation of additional mutations or selection of resistant clones, is a well-recognized mechanism in the development and relapse of hematological malignancies (14, 15). The study of clonal hematopoiesis through the longitudinal sampling of pre- and post-chemotherapy tissues offers the opportunity to gain a deeper understanding the stepwise process of malignant transformation in blood.

In the absence of a hematological malignancy diagnosis, genetic variants with low allele frequencies in circulating cells can drive the clonogenic transformation of hematopoietic stem cells (16, 17). The longitudinal assessment of rare variants is a fundamental step to understanding the mechanism driving therapy-related hematological malignancies and implementing screening programs for high-risk patients undergoing chemotherapy. Current analyses of low-frequency variants are mostly based on the targeted sequencing (14, 18) of a few known leukemia-related genes. Because this strategy focuses on only known genes, it does not entirely capture the complex landscape of such mutations.

To address this gap in knowledge, we designed a specific protocol for identifying CHIP variants. We performed whole-exome sequencing with unique molecular identifiers, coupled with a mutation-calling strategy that adopts 0.5% as the lower threshold for VAF in cell free DNA (cfDNA). The use of unique molecular identifiers is intended to reliably call mutations with extremely low allele frequency, thereby avoiding the use of post-sequencing bioinformatic approaches for error suppression (19). This particular approach enabled the identification of a high number of candidate CHIP mutations whose frequencies changed after neoadjuvant chemotherapy (NACT). In addition, our analysis identified CHIP mutations associated with favorable and unfavorable treatment outcomes; finally, we characterized CHIP variants in tumor specimens.

## Materials and methods

### Patients

Blood samples from 10 patients treated under a systematic surgical algorithm at The University of Texas MD Anderson Cancer Center (Houston, TX) were retrieved from the Gynecologic Tumor Bank after written informed consent was obtained under a protocol approved by the Institutional Review Board (LAB10-0850), as we described previously (20, 21). These patients had upfront inoperable disease and received carboplatin-based NACT (neoadjuvant chemotherapy). After 3-4 cycles of NACT, patients were considered to have excellent response (NACT-ER) if they had a complete response or only microscopic disease at the time of interval surgery; they were considered to have poor response (NACT-PR) if they had stable or progressive disease on radiologic evaluation and/or suboptimal interval cytoreduction after NACT, according to Response Evaluation Criteria in Solid Tumors version 1.1.

### Isolation of cfDNA and WBC DNA and sequencing

Genomic DNA was extracted with the QIAamp DNA Mini Kit (Qiagen) and then quantified using the Quant-iT PicoGreen dsDNA Assay Kit (ThermoFisher Scientific); quality was assessed using Agilent High Sensitivity D5000 ScreenTape and Reagents on the TapeStation 4200 system (Agilent Technologies). cfDNA was extracted using the MiniMax High Efficiency Cell-Free DNA Isolation Kit (#A17622-50; Apostle) and then quantified using the Quant-iT PicoGreen dsDNA Assay Kit; quality was assessed using Genomic DNA ScreenTape and Reagents on the TapeStation 4200 system. Each genomic DNA sample (up to 200 ng, based on the PicoGreen quantification) was sheared (mechanically fragmented) using the E220 focused-ultrasonicator (Covaris) with the following settings: peak incident power, 200 Watts; duty cycle, 25%; cycles per burst, 50; duration, 10 seconds; iterations, 70. To ensure the proper fragment size, we examined the samples on the TapeStation 4200 system using Agilent High Sensitivity D1000 ScreenTape and Reagents. Libraries of the sheared DNA and unsheared cfDNA were prepared using the SureSelect XT_HS2 DNA Reagent Kit (Agilent) with 384 unique dual sample indexing and dual molecular barcodes to better suppress false positives and more accurately detect low VAFs. This protocol consisted of 3 enzymatic reactions for end repair, A-tailing, and adaptor ligation followed by barcode insertion by polymerase chain reaction (PCR) using Herculase II Fusion DNA Polymerase (Agilent; 8-14 cycles, based on input DNA quality and quantity). PCR primers were removed by using 1x volume of the AMPure PCR Purification kit (Agencourt Bioscience Corporation). The quality and quantity of the prepared libraries were evaluated using the High Sensitivity D1000 ScreenTape on Tapestation 4200 system to verify correct fragment size and ensure the complete removal of primer dimers. Subsequently, the prepared libraries were individually hybridized to SureSelect Human All Exon V4 probes (Agilent). The hybridization steps were automated on the Sciclone G3 NGSx Workstation (PerkinElmer, Inc.). Agilent Captured regions of interest were hybridized as single-sample reactions using 500–1000 ng of the prepared library as input. All hybridization and post-hybridization captures and washes were performed according to Agilent’s protocol. Briefly, the capture reagents and probes were added to the prepared libraries, and the mixture was incubated at 65°C in a thermocycler with a heated lid for up to 24 hours. The targeted regions were captured using streptavidin beads, the streptavidin-biotin-probe-target complex was washed, and the captured libraries were enriched by PCR amplification according to the Agilent’s protocol. The quality and quantity of each captured sample were analyzed on the TapeStation 4200 system using the DNA High Sensitivity Kit. The captured libraries were sequenced on the Illumina NovaSeq 6000 platform for 2 × 150 paired-end reads with an 8-nt read for indexes using Cycle Sequencing v1.5 reagents (Illumina). Demultiplexing was performed using Illumina’s bcl2fastq or BCL Convert software to generate paired-end reads based on the dual indexes and remove sequences with incorrectly paired P5 and P7 indexes. The Agilent Genomics NextGen Toolkit (AGeNT) was used for molecular barcode extraction and trimming.

### CHIP mutation calling strategy and statistical analyses

Sequencing output fastq files were first processed with fastp (version 0.23.0) using parameters –disable_adapter_trimming, –qualified_quality_phred 20, –unqualified_percent_limit 30, and –average_qual 20. Processed reads were then mapped to human genome hg38 using the Burrows-Wheeler Aligner (version 0.7.15) (22) using command “bwa mem -Ma” and default settings. The result file is converted to BAM file using samtools (v1.9), and genome coverages were estimated using “samtools idxstats” to obtain an average coverage on each chromosome followed by calculating a median weighted coverage adjusted by chromosome sizes as reported in Agilent_Human_Exon_V4_Regions.S043800110.hg38 bed file. To minimize PCR introduced sequencing error, we edited the BAM files and collapsed reads with the same molecular barcodes. When more than two sequencing reads are mapped to a same chromosomal start and end position, they are collapsed into two reads with the same sequences which is the most occurred sequence at this position. Only reads with properly positioned molecular barcodes were handled (ca. 85%). Reads with improper barcodes or mapping were discarded. In addition, paired reads with overlapping sequencing area are clipped on the forward strand to avoid counting the same sequence twice. The duplication percentages of original and UMI collapsed BAM files were reported by Picard MarkDuplicate function (Picard v2.27.4) (21). Varscan (version 2.4.2) was used to call variant SNPs on a pile up file generated by samtools with parameters –min-coverage 20, –min-reads2 2, –min-avg-qual 20, –p-value 0.05, and –min-var-freq 0.005. Result variants were annotated using GATK Funcotator (v4.2.4.0, data source v1.7.20200521g) and default settings. CHIP variants were defined as heterozygous variants found in both the WBC and cfDNA samples from the same patients with VAF of less than 5% in the WBC sample and more than 0.5% in cfDNA samples. Variants not meeting these criteria were considered non-CHIP variants. Known CHIP genes (n = 50) were compiled from previous studies (2, 23). Cancer Gene Census genes (n = 733) were collected from COSMIC (https://cancer.sanger.ac.uk/census) (24). Hypergeometric tests were used to examine whether Cancer Gene Census were enriched within the CHIP and non-CHIP gene sets.

Comparative analysis of the differential CHIP variants among clinical groups (all pre-NACT samples versus all post-NACT samples; PR pre-NACT samples versus PR post-NACT samples; ER pre-NACT samples versus ER post-NACT samples) were performed using R limma package (R v4.1.0, limma v3.52.4). We considered P values less than 0.05 to indicate statistical significance. Owing to the limited number of samples, most of the adjusted p-values obtained from limma analysis did not indicate significance. We therefore used an additional Wilcoxon test to prioritize limma results for putative top differential CHIP variants. For pathway analysis IPA (ingenuity pathway analysis) from Qiagen was used.

## Results

### Patients and whole-exome sequencing

**Figure 1:**
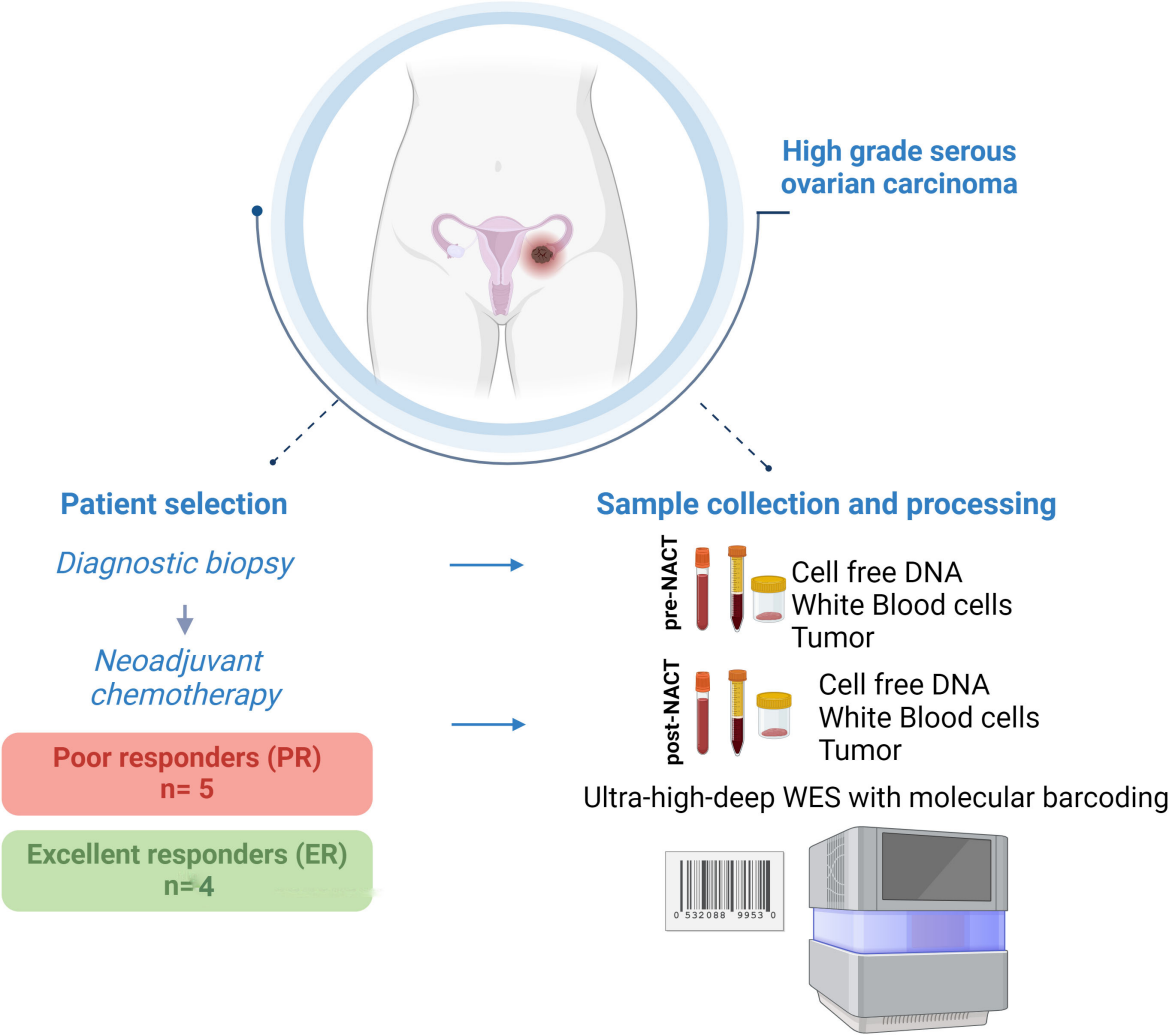
Graphical representation of the study design.

The study included 9 patients reported previously (20, 21). Of these patients, 4 had an excellent response (ER) to NACT (NACT-ER), and 5 had a poor response (PR) to NACT (NACT-PR) (**Table 1**). Isolated cfDNA, white blood cell (WBC) DNA, and tumor DNA from each patient were subjected to ultra-high-depth whole-exome sequencing using unique molecular barcodes (**Figure 1**).

**Table 1:** Clinico-pathological characteristics of the patients’ cohort.

Group	Patient ID	Age (yr)	BMI (kg/m^2^)	Race	CA-125 at diagnosis (units/ml)	Disease site	Stage	BRCA status
NACT-ER	NACT-ER-1	49	28.9	Asian	26.6	Ovarian	Stage IIIC	N.A.
	NACT-ER-6	67	38.4	Black	595.9	Ovarian	Stage IVB	N.A.
	NACT-ER-7	71	21.4	White	740.1	Ovarian	Stage IIIC	No mutation
	NACT-ER-8	78	23.9	White	365.3	Ovarian	Stage IIIC	No mutation
NACT-PR	NACT-PR-2	57	32.1	White	494.6	Peritoneum	Stage IIIC	No mutation
	NACT-PR-3	73	18.9	White	335.3	Ovarian	Stage IVB	No mutation
	NACT-PR-5	62	29.4	Hispanic	1467	Ovarian	Stage IIIC	N.A.
	NACT-PR-6	73	30.8	White	335.6	Ovarian	Stage IIIC	No mutation
	NACT-PR-10	59	19.9	White	551.3	Ovarian	Stage IVA	N.A.

NACT, neoadjuvant chemotherapy; ER, excellent response; PR, poor response; N.A., not available.

### Identification of novel candidate low-frequency CHIP variants in cf DNA and WBC DNA

We defined low-frequency candidate CHIP variants as heterozygous variants that were present in both WBC DNA and cfDNA and that had a VAF below 5% in the WBC DNA and above 0.5% in the cfDNA. We considered genes in which CHIP mutations were previously identified to be known CHIP genes (2, 23). The total number of low-frequency CHIP variants was 93,088, involving 13,780 genes (**Supplementary Table 1**). Of these variants, 463 were in 44 known CHIP genes, and 5,548 were in 598 genes listed in the Cancer Gene Census in the Catalogue of Somatic Mutations in Cancer (COSMIC) (**Supplementary Table 2A**). The total number of recurrent candidate CHIP variants present in at least 2 patients was 47,961, involving 13,509 genes. Of these variants, 266 were in 44 known CHIP genes, and 2,874 were in 585 genes listed in COSMIC (**Supplementary Table 2B**).

### Enrichment of candidate CHIP variants in post- versus pre-NACT samples by chemotherapy response

**Figure 2:**
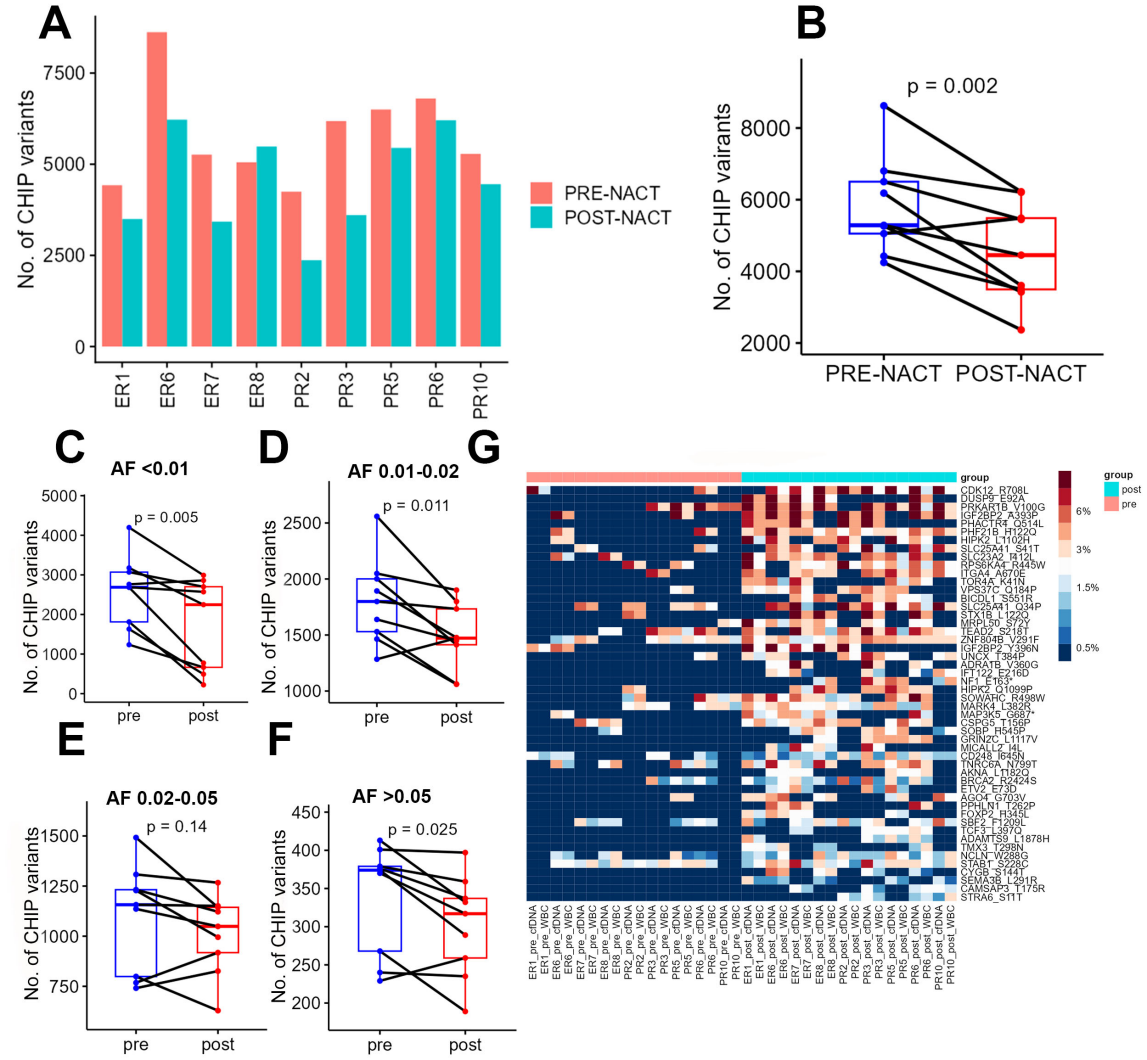
**(A)** Numbers of CHIP variants in each pre-chemotherapy sample and each post-chemotherapy sample. **(B)** Numbers of CHIP variants in all pre-chemotherapy samples versus all post-chemotherapy samples. **(C-F)** Numbers of CHIP variants with allele frequencies of 0.5-1% **(C)**, 1-2% **(D)**, 2-5% **(E)**, and more than 5% **(F)** in pre-chemotherapy samples versus post-chemotherapy samples. **(G)** Heatmap showing the distribution of CHIP variants in POST nast-NACT and in pre-NACT. pre, pre-chemotherapy; post, post-chemotherapy; var, variant; AF, allele frequency; ER, excellent response; PR, poor response; cf, circulating free DNA.

After quantifying the candidate CHIP variants in the liquid biopsy samples, we compared all patients’ pre- and post-chemotherapy samples. Post-chemotherapy samples consistently had fewer CHIP variants than pre-chemotherapy samples did (**Figures 2A, B**). To determine whether this change depended on the variant’s original allele frequency (before chemotherapy), we segregated the CHIP variants into 4 groups based on their VAFs in cfDNA in pre-chemotherapy samples (<1%, 1-2%, 2-5%, and >5%) and compared the pre- and post-chemotherapy frequencies of the CHIP variants in each group. This difference in number across groups, in terms of decrease in post-chemotherapy samples, was significant (p= 0.002, **Figure 2B**). However, after segregation, the total number of CHIP mutations whose VAFs were between 2 and 5% did not differ significantly between pre-NACT cfDNA and post-NACT cfDNA (**Figures 2C–F**).

To identify the candidate CHIP variants that were potentially selected by chemotherapy, we performed a comparative analysis of all patients’ pre- and post-chemotherapy samples to identify variants whose VAFs increased after chemotherapy. For this analysis, we first considered each CHIP variant’s VAF to be its average VAF in both WBC DNA and cfDNA. We identified 77 mutations with increased allele frequencies after chemotherapy (**Figure 2G**, **Supplementary Table 3A**), but none of these increases were statistically significant after adjustment for multiple hypothesis testing. We then sought to identify the pathways that were enriched among the genes whose CHIP variants had higher VAFs after chemotherapy. We identified enrichment in the activation of *NMDA receptors and postsynaptic events* (p = 8.93E-05; 4.7% genes overlap), *transcriptional regulation by RUNX3* (p = 1.43E-04; 4.2% genes overlap), *ERK/MAPK signaling* (p = 3.52E-04; 2.3% genes overlap), *Signaling by TGFBR3* (p = 3.54E-04; 6% genes overlap), and *transcriptional regulation by MECP2* (p =7.00E-04; 4.8% genes overlap). In addition, the genes whose CHIP mutations had higher VAFs after NACT were involved in cellular and molecular functions, including *cellular development* (p = 1.36E-02-9.64E-06), *cellular growth and proliferation* (p = 1.36E-02-.5 = 9.64E-06), *Cell Death and Survival* (p = 1.31E-02 4.6E-05- 0.00329), *gene expression* (p = 1.36E-0.2-5.99E-05), and *Cell Morphology* (p =1.24E-02- 9.52E-05). The candidate upstream regulators for some of these genes were TNKS1BP1 (p= 1.78E-05), *DNMT3A* (p = 5.95E-05) and *TET3*(p = 2.51E-04), some genes previously associated with leukemia (25, 26). The top candidate CHIP mutations included a missense mutation (p.R708L) in *CDK12*, which encodes cyclin-dependent kinase 12, a protein involved in the tumorigenesis of several cancers. To test the reliability of circulating DNA as a source material for CHIP variant quantification, we performed the same comparative analysis using only the candidate CHIP variants’ VAFs in cfDNA; of 78 variants with higher VAFs after chemotherapy, 52 were also identified in the previous analysis (**Supplementary Table 3B**, **Supplementary Figure 1A**).

**Figure 3:**
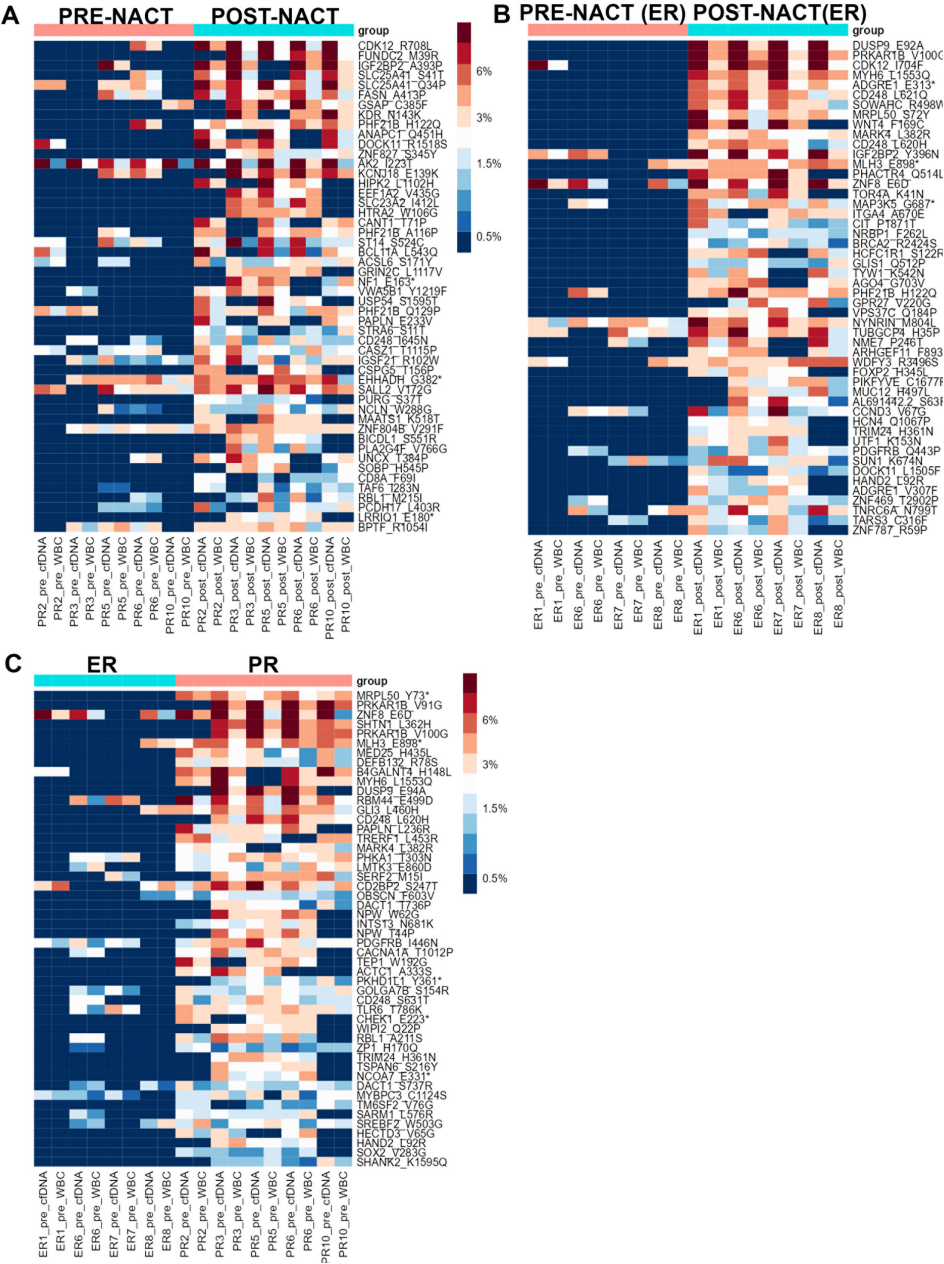
**(A)** Heatmap showing the distribution of the top 50 candidate CHIP mutations whose VAFs were higher in post-NACT samples than in PR pre-NACT samples in the PR group. **(B)** Heatmap showing the distribution of the top 50 candidate CHIP mutations whose VAFS were higher in post-NACT samples than in pre-NACT samples in the ER group. **(C)** Heatmap showing the distribution of the top 50 candidate CHIP mutations whose VAFs in pre-NACT samples were higher in the PR group than in the ER group. Asteriscs indicate stop codons.

We then sought to determine whether CHIP variants’ VAFs differed between pre- and post-NACT liquid biopsy samples for patients with NACT-ER and for patients with NACT-PR. For PR patients, 85 CHIP mutations had higher VAFs in post-NACT samples than in pre-NACT samples, but none of the differences was statistically significant (**Figure 3A**, **Supplementary Table 4A**). For ER patients, 2 candidate CHIP mutations had significantly higher VAFs after NACT: p.V100G, in *PRKAR1B* (adj. p = 0.001845171); p.E92A, in *DUSP9* (adj. p = 0.008174934) (**Figure 3B**, **Supplementary Table 5A**). We then compared CHIP variants’ VAFs in cfDNA before and after chemotherapy. In PR samples, 79 variants with higher VAFs after chemotherapy (**Supplementary Table 4B**, **Supplementary Figure 1B**). In ER samples 75 variants had higher allele frequency after chemotherapy (**Supplementary Table 5B**, **Supplementary Figure 1C**).

A comparison of CHIP variants’ VAFs in post-NACT samples from NACT-PR and NACT-ER patients revealed that 59 variants had higher VAFs in PR samples than in ER samples (**Supplementary Table 6A**, **Supplementary Figure 2A**). A comparison of CHIP variants’ allele frequencies in post-NACT cfDNA samples from ER and PR patients revealed that 45 variants had higher allele frequencies in PR samples; of these, 28 were also identified in the previous analysis, but these differences were not statistically significant (**Supplementary Table 6B**, **Supplementary Figure 2B**).

### Enrichment of candidate CHIP variants in pre-NACT samples from patients with NACT-PR

To identify novel prognostic markers that can be detected with non-invasive techniques (such as liquid biopsies) in ovarian cancer patients, we sought to identify candidate CHIP variants whose frequencies were higher in pre-NACT samples from NACT-PR patients than those from NACT-ER patients (**Figure 3C**). Our analysis identified 1 CHIP variant, p.Y73*, in *MRPL50* (adj. p = 0.018876) (**Supplementary Table 7A**). *MRPL50* encodes mitochondrial ribosomal protein L50, and the detected variant is not a previously known CHIP variant or in the COSMIC database. A comparison of CHIP variants’ allele frequencies in pre-NACT cfDNA samples from PR and ER patients revealed 70 variants whose allele frequencies were higher in PR samples (**Supplementary Table 7B**, **Supplementary Figure 2C**).

### Identification of candidate CHIP variants in tumor tissues

**Figure 4:**
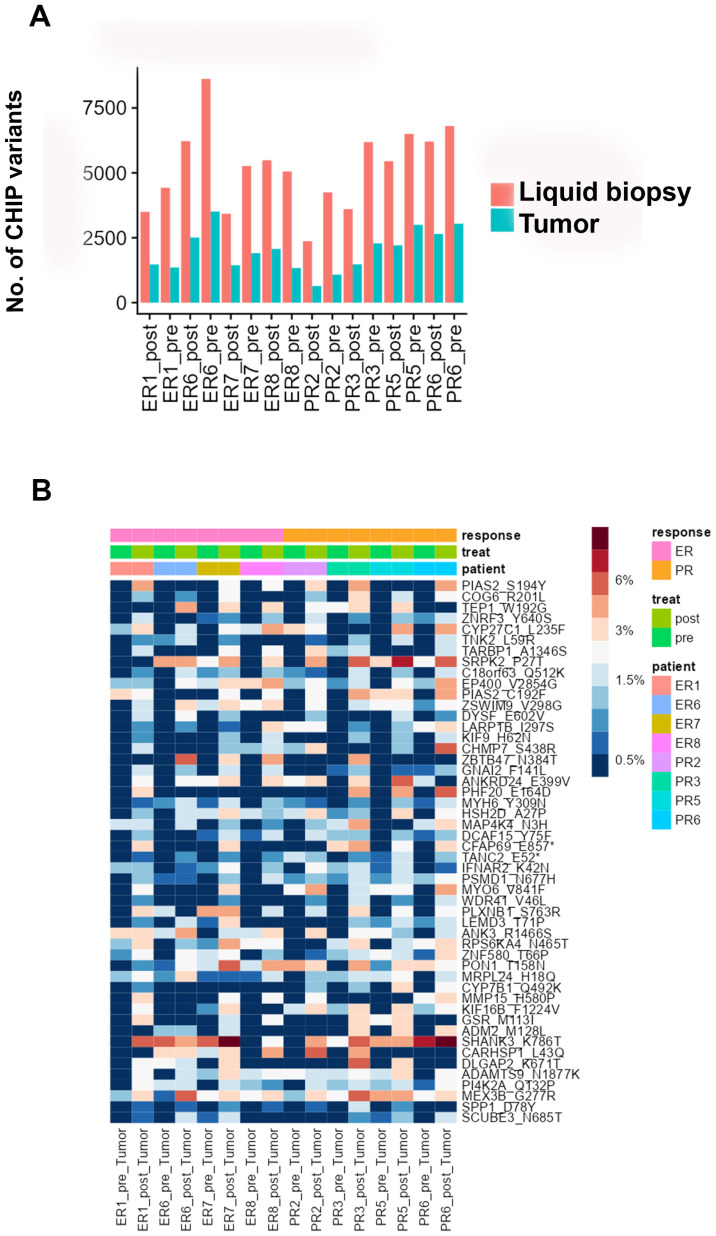
**(A)** Numbers of CHIP variants in cfDNA and tumor DNA in each pre-chemotherapy sample and each post-chemotherapy sample. **(B)** Heatmap showing the distribution of the top 50 candidate CHIP mutations whose VAFs were higher in pre-NACT samples than in post-NACT tumor samples.

To investigate the intra-tumoral presence of clonal hematopoietic cells characterized by CHIP mutations, we used deep sequencing to analyze DNA from pre- and post-NACT tumor samples matched to the cfDNA and WBC DNA. We found that 26-46% of the CHIP mutations in cfDNA matched those in tumor DNA (**Figure 4A**, **Supplementary Table 8**). The LIMMA test identified several candidate CHIP mutations whose VAFs were higher in post-NACT tumor DNA than in pre-NACT tumor DNA, but these differences were not significant after p-value adjustment (**Figure 4B**, **Supplementary Table 9**). Among patients with NACT-PR, 221 CHIP variants had higher allele frequencies, and 93 had lower allele frequencies, after chemotherapy (**Supplementary Tables 10A, B**). Among patients with NACT-ER, 165 CHIP variants had higher allele frequencies, and 121 had lower allele frequencies, after chemotherapy (**Supplementary Tables 11A, B**). A pathway analysis of the genes affected by variants with higher allele frequencies after chemotherapy identified the *Myelination Signaling Pathway* (p = 1.24E-04; overlap 2.7%) as the top canonical pathway.

## Discussion

In the present study, we intended to fully characterize the presence of CHIP variants with extremely low allele frequency (<1%) in the circulating DNA of a well-characterized cohort of ovarian cancer patients who received platinum-based NACT. Moreover, our application of ultra-high depth whole-exome sequencing with unique barcode identifiers enabled us to reliably identify variants with very low allele frequencies.

Our approach enabled identification of a large number of variants, many of them in cancer-related genes in the COSMIC database. These variants tended to have lower allele frequencies in post-chemotherapy samples, possibly because of the selective pressure chemotherapy applies to hematological clones, resulting in the loss of less proliferative clones characterized by low-frequency variants. When considering CHIP variants with allele frequencies between 2 and 5% in cfDNA, their abundance in post-chemotherapy samples was not significantly lower than in pre-chemotherapy samples. CHIP variants that persist in circulating DNA after chemotherapy and acquire higher allele frequency may represent mutations harbored by hematopoietic clones with the potential to give rise to malignancies. Our analysis uncovered such variants and identified several mutated genes whose candidate upstream regulators include *DNMT3A*, a gene whose mutations are known to be associated with clonal hematopoiesis (27).

The presence of CHIP is associated with the diagnosis of solid malignancies (17, 28), but less is known about the impact of CHIP variants on patient prognosis and chemotherapy response. Our study population provided a unique opportunity to identify the impact of CHIP mutations on chemotherapy response, due mainly to the homogeneous chemotherapy type and number of cycles. We found that a mutation in *MRPL50* (p.Y73) had a significantly higher allele frequency in pre-NACT samples from patients with NACT-PR than in those from patients with NACT-ER. This mutation was not previously identified as being associated with CHIP, and because it is not in the COSMIC database, it would be excluded from the most common panels used to screen for CHIP variants.

Because hematological cells permeate all tissues, immature hematologic clones have been detected in the microenvironments of solid tumors (17, 29). The heterogeneity of most previous studies’ patient cohorts and cancer subtypes might explain why such a phenomenon has not yet been thoroughly investigated. In the present study, therefore, we selected a highly characterized cohort of patients with the same diagnosis and similar clinical features, which enabled us to analyze matched tumor samples acquired before and after NACT. Of the CHIP variants identified in these patients’ circulating DNA, 22-46% were also present in their matched tumor tissues. What the presence of such hematological lineage–associated variants means in the context of the tumor microenvironment is unclear; prior studies speculated that these variants might derive from intratumoral “immature” immune cells and have a role in local immunosuppression (30). This is of particular interest in ovarian cancer, a solid tumor with a notoriously “cold” immune connotation, for which immunotherapy has a low success rate (31). The positive association between a high abundance of T cells and favorable prognosis (32) is evidence that immune stimulation may have a beneficial effect in ovarian cancer. A deeper screening of tumor-infiltrating immature hematological clones might offer novel insight in the immune suppressive microenvironment of ovarian cancers. Our novel findings need further validation in larger studies enrolling patients diagnosed with t-MDS or t-AML and in studies assessing the biological relevance of our candidate CHIP mutations *in vitro* and *in vivo*.

In summary, our study shows an innovative and comprehensive approach for the in-depth study of candidate CHIP variants with low allele frequency that are enriched after chemotherapy in ovarian cancer patients. This approach enabled us to identify previously unknown CHIP variants that have possible clinical implications for the development of therapy-associated hematological malignancies and the prognosis of the underlying solid tumor. These novel CHIP variants could be included in screening panels for the longitudinal follow-up of patients undergoing chemotherapy and could be used to stratify patients by their risk of secondary malignancies.

## Data Availability

The authors will provide access to data under request; the sequencing data are deposited in the EGA European Genome-Phenome Archive, dataset ID: EGAC00001001288.

## Supplementary material

Supplementary Figure 1
**(A)** Heatmap showing the distribution of CHIP mutations whose VAFs in cfDNA were higher in post-NACT samples than in pre-NACT samples. **(B)** Heatmap showing the distribution of candidate CHIP mutations whose VAFs in cfDNA were higher in post-NACT samples than in pre-NACT samples in the PR group. **(C)** Heatmap showing the distribution of candidate CHIP mutations whose VAFs in cfDNA were higher in post-NACT samples than in pre-NACT samples in the ER group.

Supplementary Figure 2
**(A)** Heatmap showing the distribution of the top 50 candidate CHIP mutations whose VAFs in cfDNA in post-NACT samples were higher in the PR group than in the ER group. **(B)** Heatmap showing the distribution of candidate CHIP mutations whose VAFs in cfDNA in pre-NACT samples were higher in the PR group than in the ER group.

Supplementary Table 1 Total CHIP variants detected.

Supplementary Table 2 Identification of novel candidate CHIP mutations. **(A)** CHIP variants identified in at least 1 patient. **(B)** CHIP variants identified in at least 2 patients.

Supplementary Table 3 CHIP variants whose average VAFs in cfDNA and WBC DNA were higher in post-NACT samples than in pre-NACT samples; b) CHIP variants whose VAFs in cfDNA were higher in post-NACT samples than in pre-NACT samples.

Supplementary Table 4 CHIP variations whose average VAFs in cfDNA and WBC DNA were higher in post-NACT samples than in pre-NACT samples, in the PR group; b) CHIP variations whose VAFs in cfDNA were higher in post-NACT samples than in pre-NACT samples, in the PR group.

Supplementary Table 5
**(a)** CHIP variations whose average VAFs in cfDNA and WBC DNA were higher in post-NACT samples than in pre-NACT samples, in the ER group; **(b)** CHIP variations whose VAFs in cfDNA were higher in post-NACT samples than in pre-NACT samples, in the ER group.

Supplementary Table 6
**(a)** CHIP variations whose average VAFs in cfDNA and WBC DNA in post-NACT samples were higher in the PR group than in the ER group; **(b)** CHIP variations whose VAFs in cfDNA in post-NACT samples were higher in the PR group than in the ER group.

Supplementary Table 7
**(a)** CHIP variants whose average VAFs in cfDNA and WBC DNA s in pre-NACT samples were higher in the PR group than in the ER group; **(b)** CHIP variants whose VAFs in cfDNA in pre-NACT samples were higher in the PR group than in the ER group.

Supplementary Table 8 Percentage of overlap of CHIP variants in cfDNA and in matched tumor DNA.

Supplementary Table 9 CHIP variations whose VAFs in tumor DNA were lower in post-NACT samples than in pre-NACT samples.

Supplementary Table 10
**(a)** CHIP variations whose VAFs in tumor DNA were higher in post-NACT samples than in pre-NACT samples, in the PR group; **(b)** CHIP variations whose VAFs in tumor DNA were lower in post-NACT samples than in pre-NACT samples, in the PR group.

Supplementary Table 11
**(a)** CHIP variations whose VAFs in tumor DNA were higher in post-NACT samples than in pre-NACT samples, in the ER group; **(b)** CHIP variations whose VAFs in tumor DNA were lower in post-NACT samples than in pre-NACT samples, in the ER group.

Supplementary Table 12 Number of reads (before and after UMI collapse), median coverage, and Picard MarkDuplicate duplication percentages (before and after UMI collapse).
