# Supplementary figures and images for: Longitudinal genomic profiling of chemotherapy-related CHIP variants in patients with ovarian cancer
Source: Front Oncol. 2025 Apr 29;15:1538446. doi: 10.3389/fonc.2025.1538446 (PMC12069037; doi:10.3389/fonc.2025.1538446)

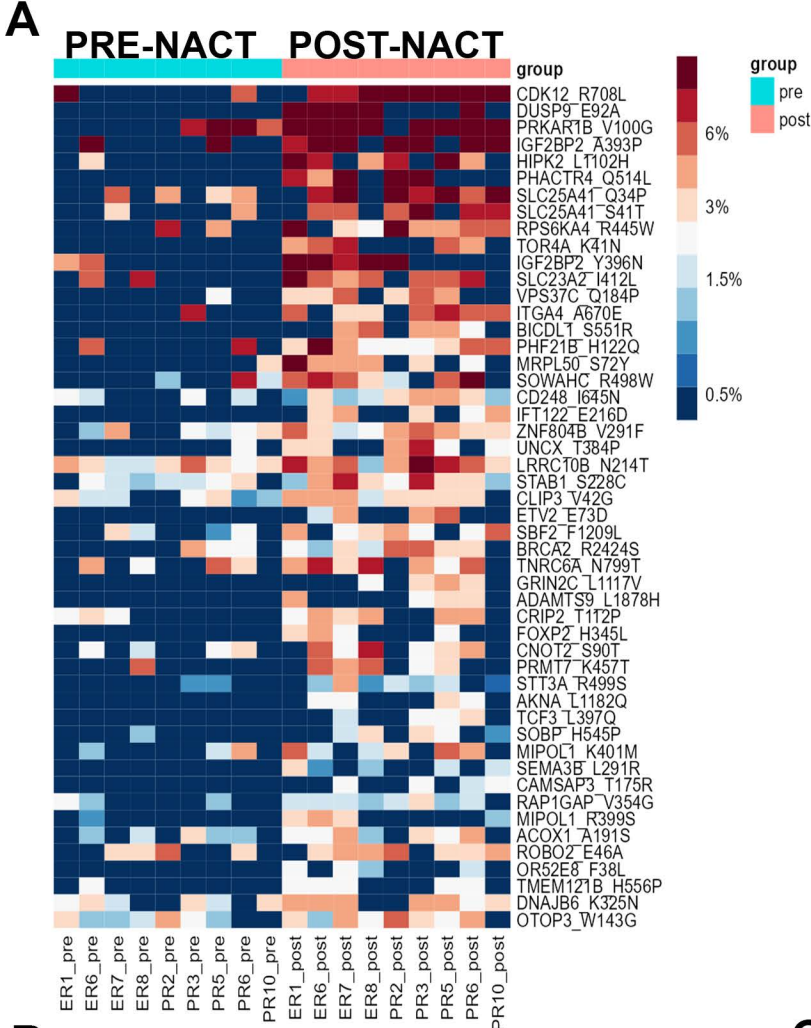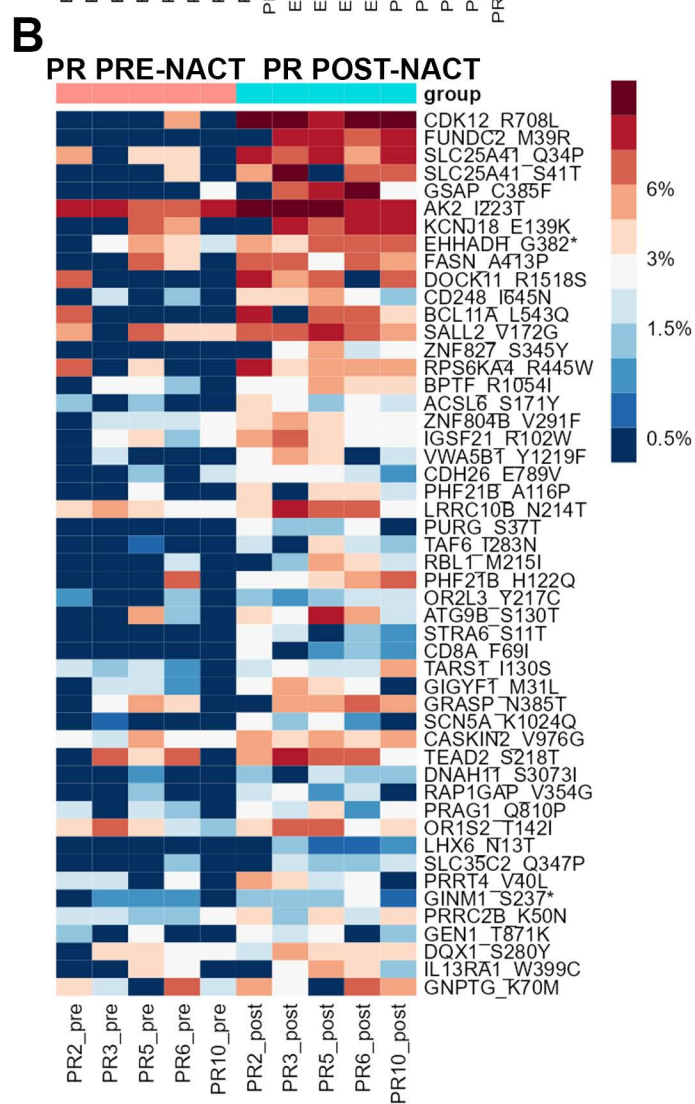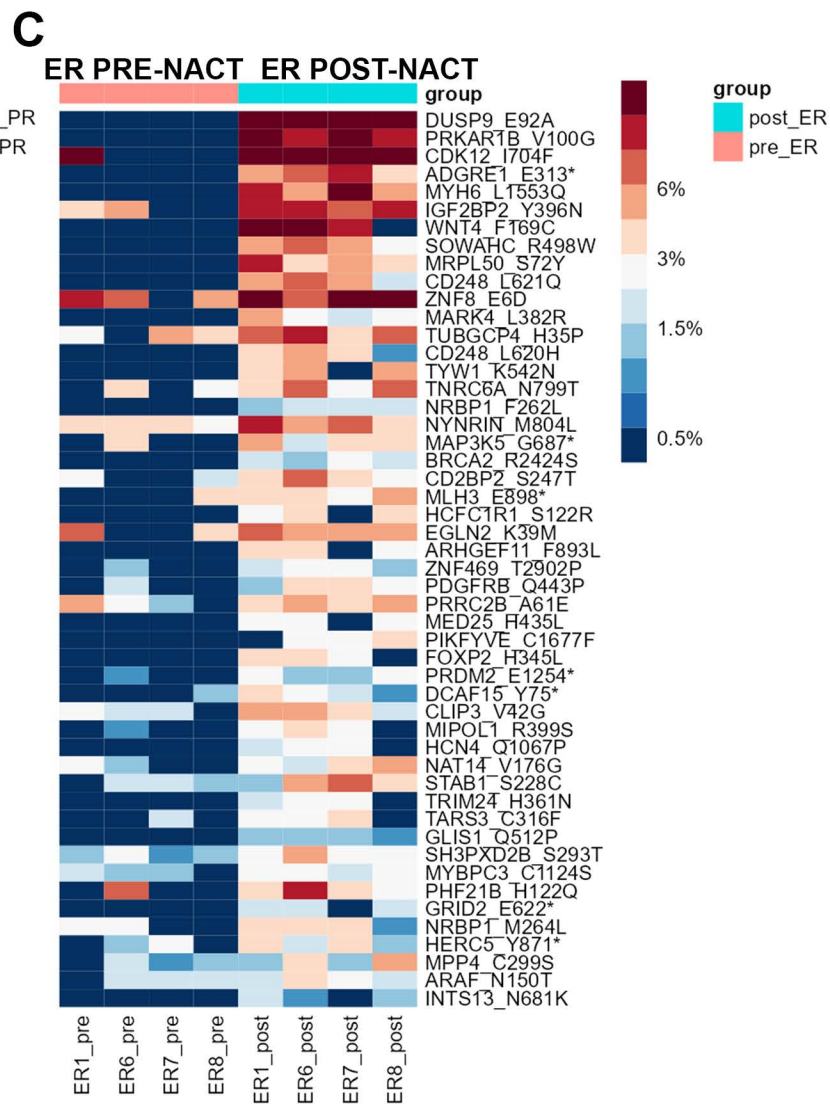

Supplement: Supplementary Figure 1 — (A) Heatmap showing the distribution of CHIP mutations whose VAFs in cfDNA were higher in post-NACT samples than in pre-NACT samples. (B) Heatmap showing the distribution of candidate CHIP mutations whose VAFs in cfDNA were higher in post-NACT samples than in pre-NACT samples in the PR group. (C) Heatmap showing the distribution of candidate CHIP mutations whose VAFs in cfDNA were higher in post-NACT samples than in pre-NACT samples in the ER group. [file Image1.pdf]

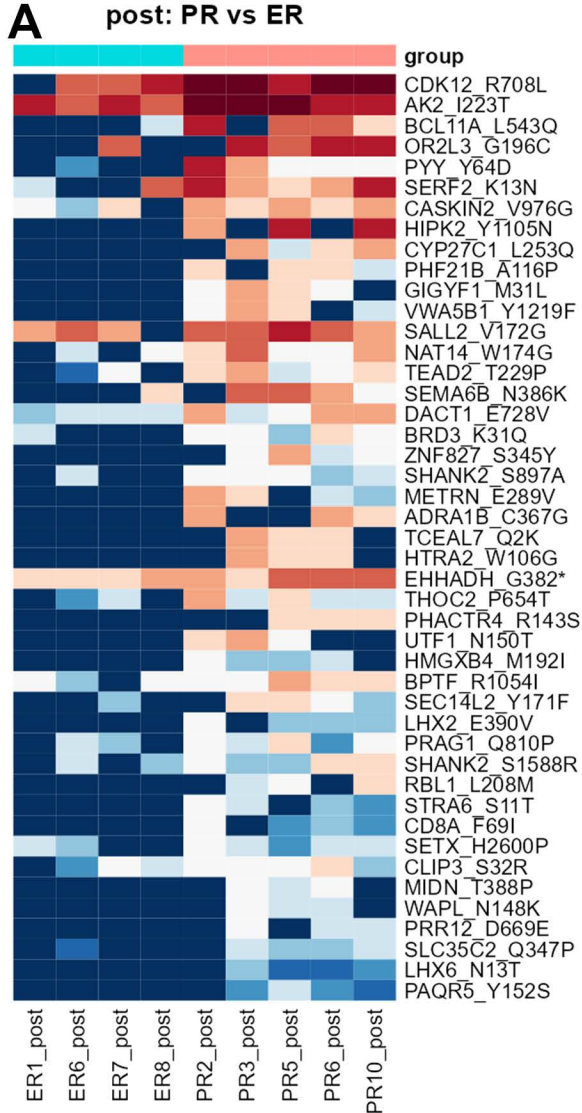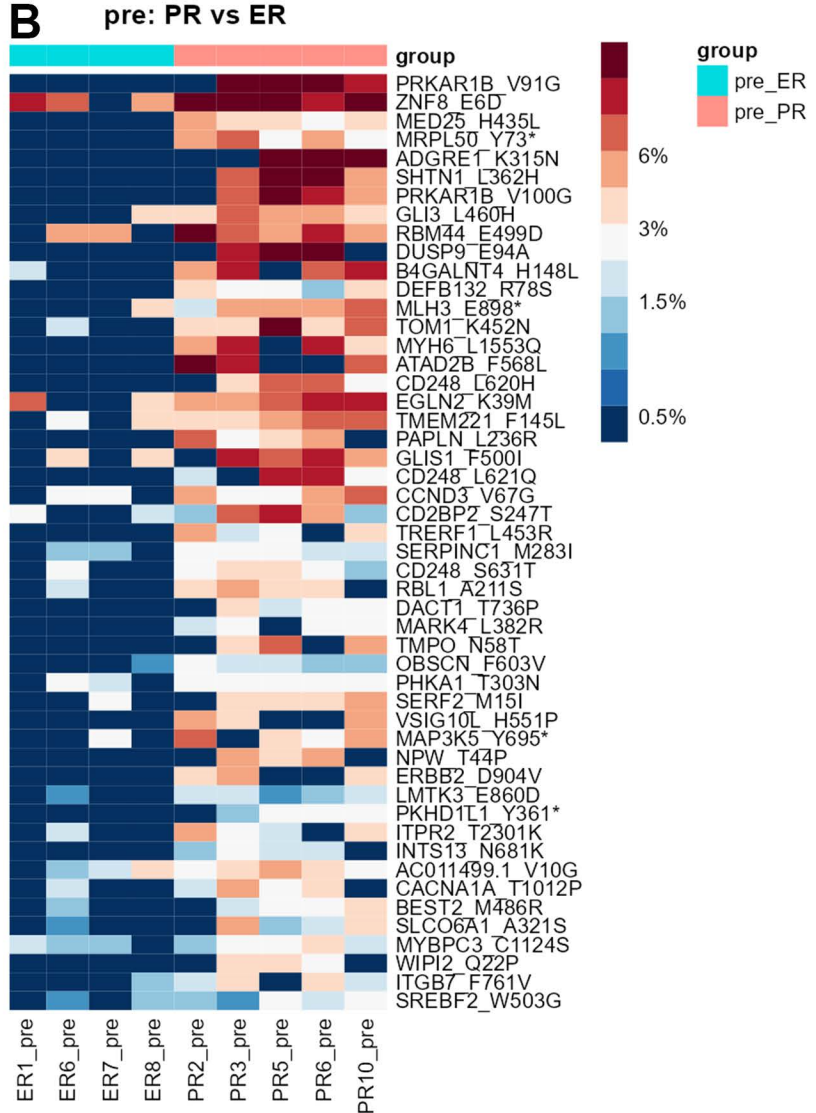

Supplement: Supplementary Figure 2 — (A) Heatmap showing the distribution of the top 50 candidate CHIP mutations whose VAFs in cfDNA in post-NACT samples were higher in the PR group than in the ER group. (B) Heatmap showing the distribution of candidate CHIP mutations whose VAFs in cfDNA in pre-NACT samples were higher in the PR group than in the ER group. [file Image2.pdf]
