# Supplementary material for: Longitudinal genomic profiling of chemotherapy-related CHIP variants in patients with ovarian cancer
Source: Front Oncol. 2025 Apr 29;15:1538446. doi: 10.3389/fonc.2025.1538446 (PMC12069037; doi:10.3389/fonc.2025.1538446)
Supplement: Supplementary Table 2 — Identification of novel candidate CHIP mutations. (A) CHIP variants identified in at least 1 patient. (B) CHIP variants identified in at least 2 patients. [file Table2.docx]

**Supplementary Table 2**: Identification of novel candidate CHIP mutations. A)Total CHIP variants identified, in at least one sample. B) CHIP variants identified in at least two patients

| **A)** | **Total** | **VAF 0.5%-1%** | **VAF 1%-2%** | **VAF 2%-5%** |
| --- | --- | --- | --- | --- |
| **Variant** | 93088 | 52216 | 25861 | 15011 |
| **Affected genes** | 13780 | 12775 | 6883 | 2775 |
| **Known CHIP variant** | 463 | 325 | 106 | 32 |
| **Known gene with CHIP** | 44 | 42 | 22 | 12 |
| **Cosmic variant** | 5548 | 3163 | 1483 | 902 |
| **Cosmic gene with variant** | 598 | 567 | 366 | 144 |
|  |  |  |  |  |
| **B)** | Recurrent | **VAF 0.5%-1%** | **VAF 1%-2%** | **VAF 2%-5%** |
| **Variant** | 47961 | 37429 | 8816 | 1716 |
| **Affected genes** | 13509 | 12449 | 5650 | 1359 |
| **Known CHIP variant** | 266 | 222 | 30 | 14 |
| **Known gene with CHIP** | 44 | 42 | 17 | 10 |
| **Cosmic variant** | 2874 | 2260 | 513 | 101 |
| **Cosmic gene with variant** | 585 | 550 | 309 | 78 |

Legend: VARIANT= novel CHIP variant; KNOWN CHIP VARIANT= known CHIP mutations present in our data set; KNOWN CHIP GENE= known genes affected by CHIP mutations present in our data set; KNOWN COSMIC VARIANT= known COSMIC variants present in our data set; KNOWN COSMIC GENE= known genes affected by COSMIC variants present in our data set; AF= allele frequency

*2014 NEJM, “Age-Related Clonal Hematopoiesis Associated with Adverse Outcomes”; 2020 JCO Precision Oncology, “Clonal Hematopoiesis in Late-Stage Non–Small-Cell Lung Cancer and Its Impact on Targeted Panel Next-Generation Sequencing.”
