# Supplementary material for: Longitudinal genomic profiling of chemotherapy-related CHIP variants in patients with ovarian cancer
Source: Front Oncol. 2025 Apr 29;15:1538446. doi: 10.3389/fonc.2025.1538446 (PMC12069037; doi:10.3389/fonc.2025.1538446)
Supplement: Supplementary Table 8 — Percentage of overlap of CHIP variants in cfDNA and in matched tumor DNA. [file Table8.docx]

**Supplementary Table 8:** Percentage of overlap of CHIP variants present in cfDNA and in matched tumor DNA.

| **Sample** | **CHIP_cfDNA** | **CHIP_tumor** | **%** |
| --- | --- | --- | --- |
| **ER1_post** | 3497 | 1470 | 42 |
| **ER1_pre** | 4424 | 1355 | 30.6 |
| **ER6_post** | 6222 | 2508 | 40.3 |
| **ER6_pre** | 8622 | 3512 | 40.7 |
| **ER7_post** | 3424 | 1439 | 42 |
| **ER7_pre** | 5265 | 1906 | 36.2 |
| **ER8_post** | 5485 | 2070 | 37.7 |
| **ER8_pre** | 5054 | 1332 | 26.4 |
| **PR2_post** | 2367 | 639 | 27 |
| **PR2_pre** | 4247 | 1081 | 25.5 |
| **PR3_post** | 3604 | 1474 | 40.9 |
| **PR3_pre** | 6183 | 2285 | 37 |
| **PR5_post** | 5448 | 2205 | 40.5 |
| **PR5_pre** | 6502 | 2998 | 46.1 |
| **PR6_post** | 6205 | 2646 | 42.6 |
| **PR6_pre** | 6802 | 3040 | 44.7 |
